# Digoxin Use and Adverse Outcomes in Patients With Atrial Fibrillation

**DOI:** 10.1097/MD.0000000000002949

**Published:** 2016-03-25

**Authors:** Wu-Tao Zeng, Zhi-Hao Liu, Zhu-Yu Li, Ming Zhang, Yun-Jiu Cheng

**Affiliations:** From the Department of Cardiology (W-TZ, Y-JC), the Eastern Hospital of the First Affiliated Hospital, Sun Yat-Sen University, Guangzhou, China; Department of Emergency (Z-HL), the First Affiliated Hospital, Sun Yat-Sen University, Guangzhou, China; Department of Obstetrics and Gynecology (Z-YL), the First Affiliated Hospital, Sun Yat-Sen University, Guangzhou, China; Department of Cardiology (MZ), Beijing Anzhen Hospital, Capital Medical University, Beijing, China.

## Abstract

Digoxin has long been used for rate control in atrial fibrillation (AF); its safety remains controversial.

We performed a literature search using MEDLINE (source PubMed, January 1, 1966, to July 31, 2015) and EMBASE (January 1, 1980, to July 31, 2015) with no restrictions. Studies that reported relative risk (RR) estimates with 95% confidence intervals (CIs) for the associations of interest were included. Pooled effect estimates were obtained by using random-effects meta-analysis.

Twenty-two studies involving 586,594 patients were identified. Patients taking digoxin, as compared with those who took no digoxin, experienced an increased risk of death from any cause (RR: 1.29[95% CI 1.16–1.43]), even after reported adjustment for propensity scores (RR: 1.28[95% CI 1.18–1.39]). The risk of death was increased with patients with or without heart failure (RR: 1.12[95% CI 1.02–1.23] and RR: 1.26[95% CI 1.15–1.29], respectively), and patients taking or not taking beta blockers (RR: 1.17 [95% CI 1.06–1.30] and RR: 1.28 [95% CI 1.08–1.51], respectively). Digoxin use was also associated with increased risk of cardiovascular death (RR: 1.32 [95% CI 1.07–1.64]), arrhythmic death (RR: 1.38 [95% CI 1.07–1.79]), and stroke (RR: 1.20 [95% CI 1.004–1.44]). Digoxin treatment is associated with an absolute risk increase of 19 (95% CI 13–26) additional deaths from any cause per 1000 person-years.

Digoxin use is associated with a significant increased risk for death from any cause in patients with AF. This finding suggests a need for reconsideration of present treatment recommendations on use of digoxin in AF.

## INTRODUCTION

Atrial fibrillation (AF) is the most prevalent cardiac arrhythmia encountered in clinical practice, affecting >33.5 million individuals worldwide.^1^ AF increases the risk of stroke, congestive heart failure (HF), ventricular arrhythmias, and death. A key treatment target in AF is heart rate control, which might help reduce symptoms and the risk of HF.^2^ During the past 2 centuries, digoxin remains one of the most widely used rate control agent worldwide and is largely accepted as a valid therapeutic option for AF. Current American Heart Association, American College of Cardiology, and Heart Rhythm Society treatment guidelines for the management of AF recommend the use of digoxin alone for resting heart rate control in sedentary individuals (Class I recommendation, level of evidence C).^1^

Although considered generally safe, several recent studies have reported digoxin to have potential proarrhythmic properties, long-term effects on cardiac remodeling, and even link to adverse prognosis in AF.^3,4^ Actually, evidence from large cohort studies and post-hoc analyses of randomized clinical trials (RCTs) to assess a potential increase in serious cardiac events associated with digoxin is conflicting. Some studies have found no significant association between digoxin use and mortality,^5,6^ whereas other studies recently showed that digoxin was associated with increased risk for death from any cause and cardiovascular death in patients with AF ^7–9^. Interpretation of the evidence has been complicated by populations with different baseline characteristics (e.g., men vs women; concurrent with HF versus without HF; concomitant use of beta blockers vs no use of beta blockers; baseline use of digoxin vs incident use of digoxin), and different studies types (retrospective cohort studies vs prospective cohort studies versus RCTs). There is therefore a clear imperative to define the place of digoxin in the clinical management of AF and to guide physicians and patients with an indication for treatment with digoxin. Thus, we conducted a meta-analysis to examine the link between digoxin and adverse outcomes, including death from any cause, cardiovascular death, arrhythmic death, and stroke.

## METHODS

### Search Strategy

We undertook a meta-analysis of the published work without language restrictions according to the PRISMA guidelines (Appendix Text 1). We selected relevant studies published in the electronic databases MEDLINE (source PubMed, January 1, 1966, to December 31, 2015) and EMBASE (January 1, 1980, to December 31, 2015) with the following combined text and MeSH heading search strategy: “digoxin,” “mortality,” “death,” “cardiovascular,” “cardiac,” “atrial fibrillation.” We also manually scrutinized the references of all relevant articles to supplement our search.

### Study Selection

Studies were considered eligible if they met the following criteria: (1) the study designs were cohort studies, case-control studies or RCTs (animal studies, cross-sectional studies, reviews, commentaries, letters, and studies that examined other associations were excluded); (2) the outcome of interest was death from any cause, cardiovascular death, arrhythmic death or stroke; and (3) relative risk (RR) and the corresponding 95% confidence interval (CI) (or data to calculate them) were reported; (4) studies were independent. We reviewed each publication and only the most recent or complete study was included when multiple reports on the same population or subpopulation were identified.

### Data Abstraction and Quality Assessment

Three authors (WTZ, ZHL, ZYL) extracted the following information from each study: study's characteristics (study design, first author's name, publication year, geographical area, duration of follow-up, sample size and numbers of incident cases), participants’ characteristics (mean age and gender category) and analysis strategy (statistical models, confounders adjusted for, effect sizes and 95% CIs). Methodological Index for Non-Randomized Studies (MINORS) was used to evaluate the quality of all studies. Studies received 0 point to 2 points for each of these 12 components. Total score ranged from 0 point to 24 points. Studies were defined to be low-quality and high-quality studies based on their MINORS scores of <16 and ≥16 points.^10^

### Statistical Analysis

The RRs were used as the common measure of association across studies. Summary RRs were estimated by pooling the study-specific estimates using random rather than fixed effects models in order to take into account the between-study heterogeneity.^11^ To assess for heterogeneity of RRs across studies, the *I*^2^ (95% CI) statistic was calculated with the following interpretation: low heterogeneity defined as *I*^2^<50%; moderate heterogeneity defined as *I*^2^ 50% to 75%; high heterogeneity defined as *I*^2^>75%.^12^ Heterogeneity was also calculated by comparing results from studies stratified according to prespecified study-level characteristics with meta-regression and subgroup analyses.^13^ Sensitivity analysis was performed to assess the effects of selected study quality. Possible publication bias was assessed by using Begg's adjusted rank correlation test and Egger's regression asymmetry tests and by visual inspection for asymmetry of a funnel plot of the natural logarithms of the effect estimates against their standard errors.^14^ We did all analyses with Stata version 11.0 (Stata Corp) and a *P* value<0.05 was considered statistically significant.

## RESULTS

### Study Selection

With the search strategy, 1309 unique citations were initially retrieved. Of these, 268 articles were considered of interest and full text was retrieved for detailed evaluation. Two hundred and twenty-one articles were excluded with no relevant outcomes, and other 25 articles were excluded because they did not provide enough data to estimate RR, leaving 22 studies for final inclusion in the meta-analysis (Appendix Figure 1).

### Study Characteristics

A total of 586,594 patients were included in 22 eligible studies, of which 62.3% were men. Seven studies were based in Europe, 7 in North America, 3 in East Asia, and 5 were multinational. There were 11 retrospective cohort studies, 6 prospective cohort studies and 5 retrospective of RCTs. Of the primary studies, 100% had described independent, consecutive sampling of their cohort. Average follow-up duration ranged from 6.0 to 56.4 months. Patients were followed up for an average of >24 months in a majority of studies (62.5%).The sizes of the cohorts ranged from 347 to 220,068, with the 3 largest studies including patients >100,000. Digoxin use was defined as baseline use in 18 studies, and as incident use in 6 studies (Appendix Table 1). Five of the 22 studies reported data on the daily digoxin dose and/or the mean digoxin plasma levels (Appendix Table 2). The endpoint of death from any cause was reported in 21 studies, cardiovascular death was reported in 8 studies, arrhythmic death was reported in 5 studies, and stroke was reported in 7 studies, respectively. Adjusted RRs could be determined for all studies, with 11 studies reporting adjusted estimates for propensity scores (Appendix Table 1). The methodological quality of the included studies was generally good, with MINORS scores >16 points in all the studies included (Appendix Table 3).

### Association With Death From any Cause

**FIGURE 1:**
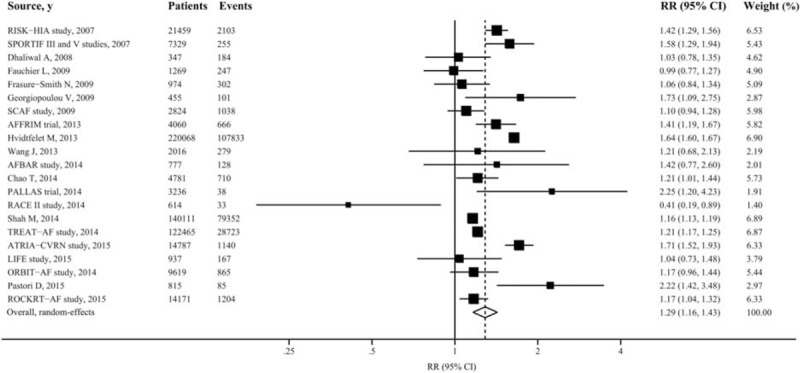
Forest plot showing relative risks of death from any cause associated with digoxin therapy in patients with atrial fibrillation. The size of each square is proportional to the study's weight (inverse of variance).

Twenty-one studies with data of 573,114 individuals and at least 225,453 events reported risk estimates for death from any cause. Among the 21 selected studies, all but 2 found an association between use of digoxin and an increased risk of death from any cause, although not all were statistically significant. Overall, patients with AF taking digoxin compared with the reference group experienced a significantly increased risk for death from any cause (RR: 1.29 [95% CI 1.16–1.43, *P*<0.001]) (Figure 1). From studies that reported information on person-years in patients taking and not taking digoxin, we could calculate absolute annual rates of death from patients with AF: 73 deaths per 1000 person-years in patients taking digoxin and 54 deaths in patients not taking digoxin, corresponding to an absolute risk increase of 19 (95% CI 13–26) deaths per 1000 person-years.

Sensitivity analysis using a fixed-effects instead of random-effects model yielded similar results (RR: 1.37 [95% CI 1.35–1.39, *P*<0.001]). In addition, risk estimates changed little after exclusion of 3 largest studies with sample size >100,000 or 2 outlier studies reporting largest RRs. Only 5 studies reported digoxin dosing and/or plasma levels, and restricting analyses to these studies showed a somewhat greater risk (RR: 1.57 [95% CI 1.09–2.27, *P* < 0.001]). Of note, when the analysis was confined to those studies with propensity matched cohort (high quality), the overall combined RR did not appreciably change (RR: 1.28 [95% CI 1.18–1.39, *P* < 0.001]) (Table 1).

**TABLE 1:**
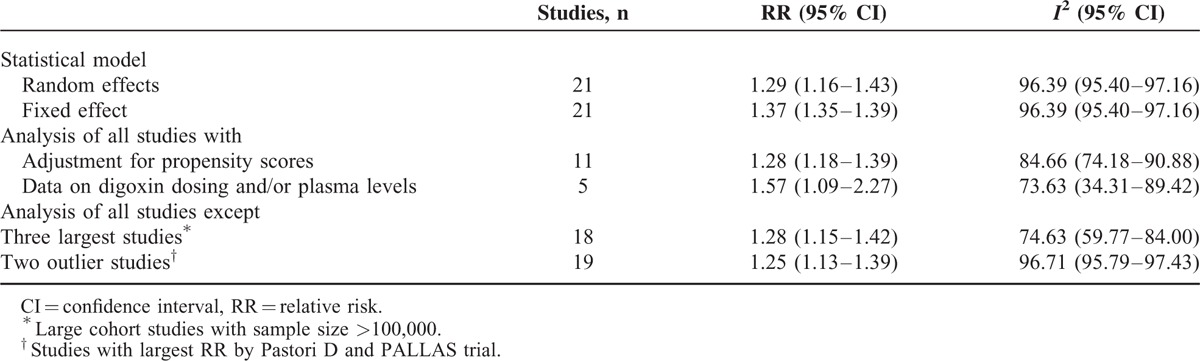
Sensitivity and Heterogeneity Analysis of Pooled Relative Risks of Death From any Cause Associated With Digoxin in Patients With Atrial Fibrillation

There was evidence of considerable heterogeneity of RRs across these studies (*I*^2^: 96.39% [95% CI 95.40–97.16%, *P* < 0.001]) (Figure 1). These measurements of heterogeneity were likely driven by the extremely large overall number of participants in our analysis (> 500,000). The point estimates of the RRs were consistently >1 in all but 2 studies, and study subgroups were more homogeneous. Neither funnel plots nor Egger and Begg tests showed evidence of publication bias (Egger, *P* = 0.10; Begg, *P* = 0.49) (Appendix Figure 2).

### Stratified Analyses

To further explore study heterogeneity, we performed stratified analyses across a number of key study characteristics and clinical factors. The finding of increased risk of death from any cause associated with digoxin was consistently observed in all of the stratified analyses. Study quality characteristics did not seem to markedly influence the results. Follow-up duration (*P* = 0.46), geographical area (*P* = 0.95), number of events (*P* = 0.56), or publication year (*P* = 0.46)) were not significantly associated with the strength of the association. Stronger associations between digoxin and risk of death from any cause were found in studies that were adjusted for 8 or more confounding factors, but these differences were not statistically significant (*P* = 0.68). Of note, similar results were observed in retrospective cohort studies (RR: 1.32 [95% CI 1.14–1.52]), prospective cohort studies (RR: 1.31 [95% CI 1.10–1.58]), and post-hoc analysis of RCTs (RR: 1.25 [95% CI 1.15–1.36]) (Table 2).

**TABLE 2:**
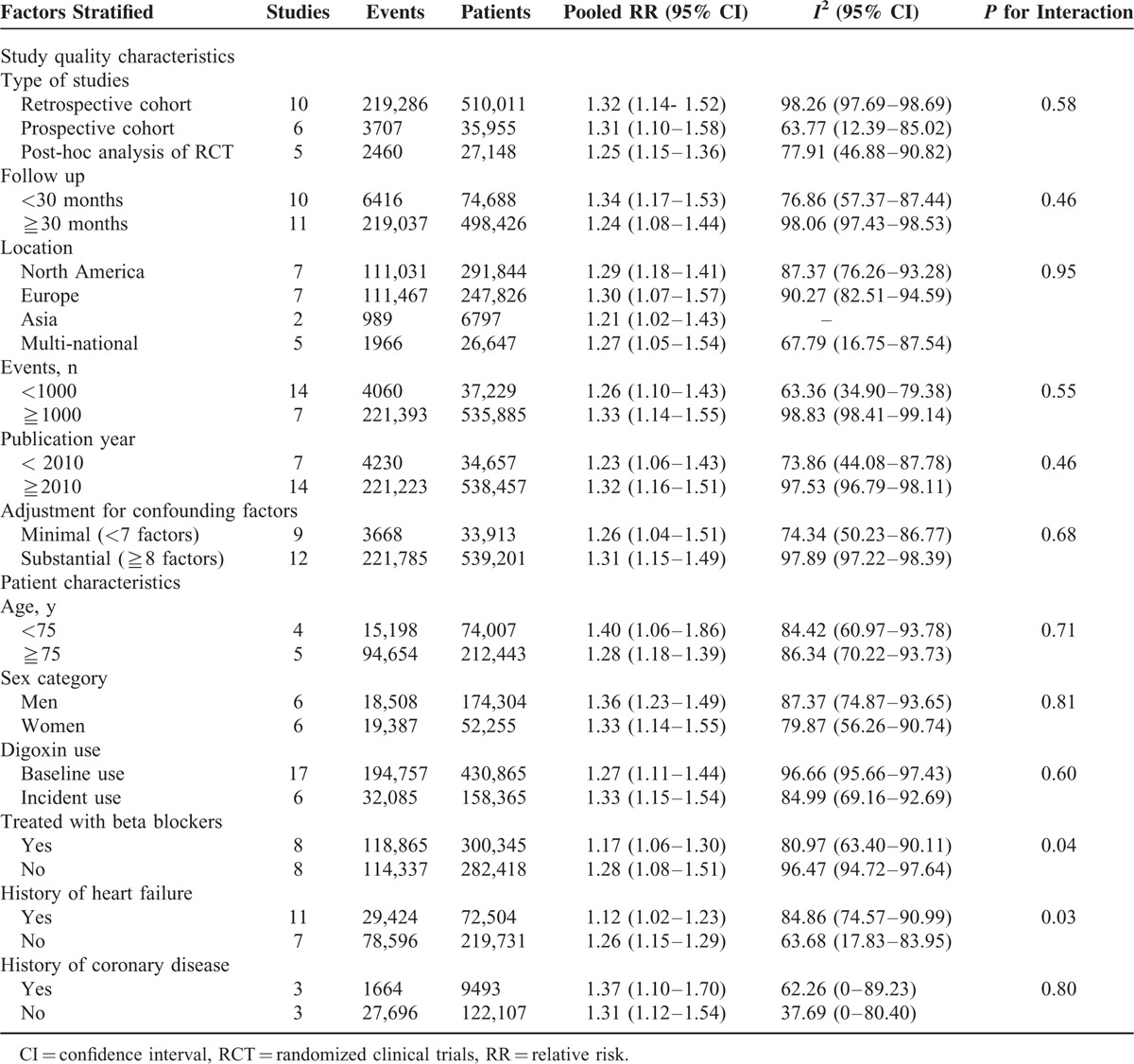
Stratified Analyses of Pooled Relative Risks of Death From any Cause Associated With Digoxin in Patients With Atrial Fibrillation

The characteristics of participants included in the primary studies seemed to be associated with the results. The association between use of digoxin and risk of death from any cause was slightly stronger in patients taking no beta blockers (RR: 1.28 [95% CI 1.08–1.51]) compared with those taking beta blockers (RR: 1.17 [95% CI 1.06–1.30])(*P* = 0.04). Furthermore, risk estimates were systematically higher in patients without HF (RR: 1.26 [95% CI 1.15–1.29]) than in patients with HF (RR: 1.12 [95% CI 1.02–1.23]) (*P* = 0.03). In studies that included both men and women, the pooled risk was similar in both sexes (RR: 1.36 [95% CI 1.23–1.49] for men and RR: 1.33 [95% CI 1.14–1.55] for women; *P* = 0.81). The association with risk of death from any cause was also similar for baseline use and incident use of digoxin (RR: 1.27 [95% CI 1.11–1.44] for baseline use and RR: 1.33 [95% CI 1.15–1.54] for incident use; *P* = 0.72), and for patients <75 years old and ≧75 years old (RR: 1.40 [95% CI 1.06–1.86] for patients <75 years old and RR: 1.28 [95% CI 1.18–1.39] for ≧75 years old; *P* = 0.71) (Table 2).

### Association With Other Adverse Outcomes

**FIGURE 2:**
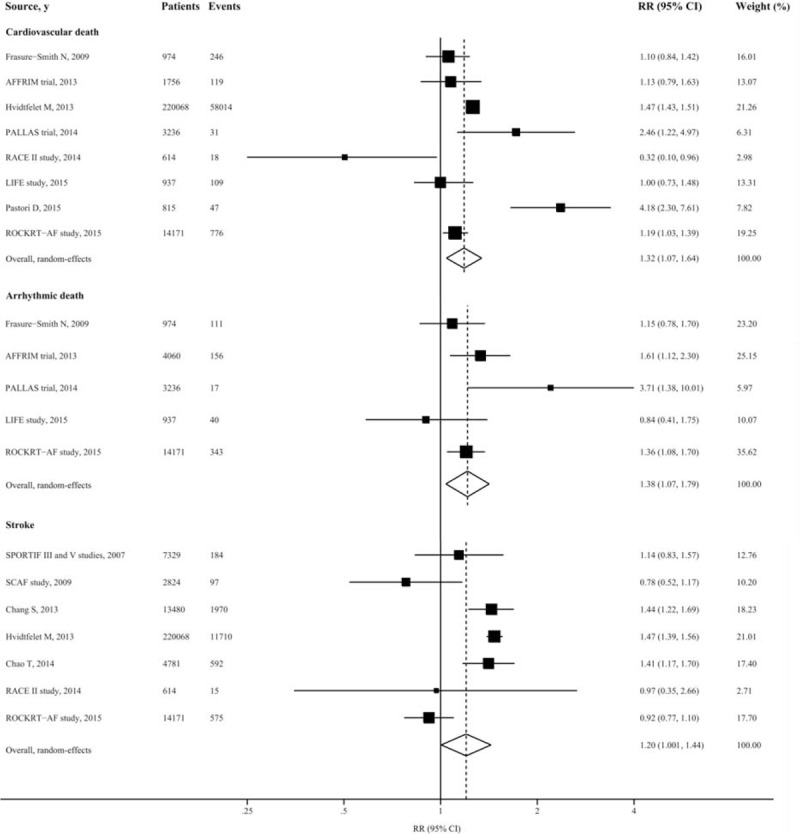
Forest plot showing relative risks of cardiovascular death, arrhythmic death, and stroke associated with digoxin therapy in patients with atrial fibrillation. The size of each square is proportional to the study's weight (inverse of variance).

For the endpoint of cardiovascular death, 8 studies were included, reporting 59,360 events among 242,571 participants. Use of digoxin was associated with increased risk of cardiovascular death in patients with AF (RR: 1.32 [95% CI 1.07–1.64, *P* = 0.009]), with evidence of high heterogeneity (*I*^2^: 82.05% [95% CI 65.82–90.58%, *P* < 0.001]) (Figure 2). However, neither funnel plots nor Egger and Begg tests showed evidence of publication bias (Egger, *P* = 0.20; Begg, *P* = 0.71).

We also had additional analysis of other end points in relation to cardiovascular disease. From 5 studies included, patients taking digoxin experienced increased risk of arrhythmic death (RR: 1.38 [95% CI 1.07–1.79, *P* = 0.01]), with no significant heterogeneity (*I*^2^: 44.29% [95% CI 0–79.56%, *P* = 0.13]). Similarly, use of digoxin was associated with increased risk of stroke for patients with AF (RR: 1.20 [95% CI 1.004–1.44, *P* = 0.045]) (Figure 2). However, digoxin did not seem to be associated with risks of HF hospitalization, acute myocardial infarction, and all cause hospitalization (Appendix Figure 3).

## DISCUSSION

The present meta-analysis, involving >500,000 participants from 22 studies, found a significantly increased risk of death from any cause associated with digoxin therapy in patients with AF, even after reported adjustment for propensity scores. These observations were consistent across all subgroups and were independent of age, sex, HF, definition of digoxin therapy, or concomitant therapy with beta-blockers. Digoxin therapy also increases risk of cardiovascular death, arrhythmic death and stroke. In terms of absolute risk, use of digoxin would account for an estimated 19 deaths per 1000 person-years. These findings challenge the current cardiovascular society guidelines, which give Class I and Class IIa recommendations for the use of digoxin as an adjunct to rate control monotherapy.^1^

There is 1 factor that may confound the interpretation of association between digoxin therapy and risk of mortality. Patients with more advanced HF, hospitalization, or more recalcitrant AF are more likely to be prescribed digoxin, which is likely to associate digoxin with worse clinical outcomes. In light of the absence of RCTs testing digoxin in patients with AF, observational studies, and post-hoc analyses of RCTs may be of particular utility for evaluating this association, despite risk of bias and confounding by indication still exists. However, in the analysis of studies with propensity matched cohort and post-hoc analyses of RCTs that provide more robust control for confounders than adjustment, the results were similar to the main findings. Of interest, we did not note increased risk with digoxin with respect to other adjudicated endpoints such as HF hospitalization, acute myocardial infarction and all cause hospitalization, reducing the likelihood of significant unmeasured confounding factors. In addition, similar results were observed in studies that focused on death associated with incident digoxin therapy to avoid biases associated with examining prevalent therapy and outcomes and captured longitudinal exposure to digoxin throughout follow-up. These indicate that risk of mortality associated with digoxin might be attributable to the prescribed treatment rather than the effect of disease severity.

The underlying mechanisms involved in the association between digoxin therapy and adverse outcomes in patients with AF are uncertain. However, several plausible explanations have been suggested. First, our results indicated that the death risk associated with digoxin might be partly mediated by its cardiovascular toxicity, supported by the evidence that risks of cardiovascular death and arrhythmic death were both increased. It is well appreciated that digoxin has a narrow therapeutic window. Freeman et al, demonstrated in the ATRIAL-CVRN study that levels of digoxin serum concentration were significantly higher in patients who died compared with those who did not die (1.515 ng/mL vs 0.935 ng/mL, *P* < 0.001).^4^ Digoxin may provoke paroxysmal atrial tachycardias and relapses of AF by promoting atrial reverse remodeling, modulating intracellular calcium signaling, and triggering oxidative stress.^3,15–18^ In addition, digoxin might also provoke serious bradycardia such as severe sinus bradycardia and high-degree atrio-ventricular block, and ventricular arrhythmias such as ventricular tachycardia, torsades de pointes, and ventricular fibrillation; these arrhythmias may be facilitated by coexistence of electrolyte imbalances (i.e., hypokalemia, hypomagnesemia, and hypercalcemia) and/or unrecognized atrio-ventricular accessory pathways.^19^ Second, it is theoretically possible for digoxin to increase noncardiac deaths, similar to the association between amiodarone and cancer deaths reported in a prior AFFIRM substudy.^20^ It is also possible that digoxin might cause noncardiac side effects such as anorexia, nausea, and vomiting and neurological disorder, and further accentuated by significant drug–drug interactions, such as clarithromycin, dronedarone, amiodarone, propafenone, or verapamil, all of which can increase serum digoxin concentrations and may increase the likelihood of digoxin toxicity.^21,22^ Third, why would digoxin therapy increase risk of stroke in patients with AF? Digoxin could inhibit the Na^+^/K^+^-ATPases, which, in turn, leads to increased levels of intracellular calcium. And activation of platelets by increased intracellular calcium levels may enhance thrombogenesis and thus the risk of ischemic stroke.^18^

Strengths of this meta-analysis include the strict inclusion criteria, the large number of patients analyzed, the robustness of the findings in sensitivity analyses, and the fact that all subgroup analyses were prespecified a priori. There are several limitations to this study. First, there is a clear and understandable discrepancy in the sample sizes from randomized and observational data. Second, there is anticipated heterogeneity of RRs among studies in the primary analysis and we therefore prespecified a random effects model, which may produce more conservative results. However, stratified analyses showed pooled RRs consistently >1 across a number of clinical factors. In addition, the consistency of the finding of an increased risk of death from any cause with digoxin therapy in both cohort studies and post-hoc analyses of RCTs suggests that the association is valid. Third, AF has a wide clinical spectrum, from asymptomatic disease to a severe uncontrolled condition. Definitions of AF in different studies varied, and we cannot exclude misclassification. Fourth, another limitation was the lack of individual participant data, which precluded determining the independent associations of individual variables with study outcomes. For example, the previous study suggested higher serum digoxin levels were significantly associated with increased mortality. Limited data on dosing or serum concentration of digoxin in original studies make it impossible to analyze the effect of high digoxin doses and serum concentrations on outcomes, including cardiovascular death and arrhythmic death. Fifth, like all meta-analyses, our study has the limitation of being a retrospective analysis.

In absolute terms, an addition of 19 deaths from any cause occurred per 1000 patients per year associated with digoxin in this study. Given that the absolute risk is not small and that digoxin remains commonly used for the heart rate control in patients with AF around the world, the total number of excess deaths may not be negligible. This calls for large well-designed RCTs of dose-adjusted digoxin therapy in AF patients. In light of this finding and the availability of other drugs for rate control in patients with AF, such as beta-blockers and nondihydropyridine calcium channel antagonists, digoxin treatment might be used with caution in patients with AF until such proper RCTs are being completed.

In conclusion, the results from this meta-analysis suggest that in patients with AF, treatment with digoxin was independently associated with increased risk of death from any cause, as well as cardiovascular death, arrhythmic death, and stroke. These results were consistent across strata of age, sex, HF status, or concomitant therapies. Given other available rate control options, digoxin might be used with caution in the management of AF until large well-designed RCTs are being completed.

## Supplementary Material

Supplemental Digital Content
