# Supplementary material for: Digoxin Use and Adverse Outcomes in Patients With Atrial Fibrillation
Source: Medicine (Baltimore). 2016 Mar 25;95(12):e2949. doi: 10.1097/MD.0000000000002949 (PMC4998364; doi:10.1097/MD.0000000000002949)
Supplement: Supplemental Digital Content [file medi-95-e2949-s001.docx]

**PRISMA 2009 Checklist**


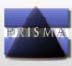


| **Section/topic** | **#** | **Checklist item** | **Reported paragraph # (within section)** |
| --- | --- | --- | --- |
| **TITLE** | | |  |
| Title | 1 | Identify the report as a systematic review, meta-analysis, or both. | **meta-analysis** |
| **ABSTRACT** | | |  |
| Structured summary | 2 | Provide a structured summary including, as applicable: background; objectives; data sources; study eligibility criteria, participants, and interventions; study appraisal and synthesis methods; results; limitations; conclusions and implications of key findings; systematic review registration number. | **Abstract was structured in format required by JAMA Internal Medicine.** |
| **INTRODUCTION** | | |  |
| Rationale | 3 | Describe the rationale for the review in the context of what is already known. | **1-2** |
| Objectives | 4 | Provide an explicit statement of questions being addressed with reference to participants, interventions, comparisons, outcomes, and study design (PICOS). | **2** |
| **METHODS** | | |  |
| Protocol and registration | 5 | Indicate if a review protocol exists, if and where it can be accessed (e.g., Web address), and, if available, provide registration information including registration number. | **N/A** |
| Eligibility criteria | 6 | Specify study characteristics (e.g., PICOS, length of follow-up) and report characteristics (e.g., years considered, language, publication status) used as criteria for eligibility, giving rationale. | **1** |
| Information sources | 7 | Describe all information sources (e.g., databases with dates of coverage, contact with study authors to identify additional studies) in the search and date last searched. | **1** |
| Search | 8 | Present full electronic search strategy for at least one database, including any limits used, such that it could be repeated. | **1** |
| Study selection | 9 | State the process for selecting studies (i.e., screening, eligibility, included in systematic review, and, if applicable, included in the meta-analysis). | **2** |
| Data collection process | 10 | Describe method of data extraction from reports (e.g., piloted forms, independently, in duplicate) and any processes for obtaining and confirming data from investigators. | **3** |
| Data items | 11 | List and define all variables for which data were sought (e.g., PICOS, funding sources) and any assumptions and simplifications made. | **N/A** |
| Risk of bias in individual studies | 12 | Describe methods used for assessing risk of bias of individual studies (including specification of whether this was done at the study or outcome level), and how this information is to be used in any data synthesis. | **4** |
| Summary measures | 13 | State the principal summary measures (e.g., risk ratio, difference in means). | **4** |
| Synthesis of results | 14 | Describe the methods of handling data and combining results of studies, if done, including measures of consistency (e.g., I^2^) for each meta-analysis. | **4** |

Page 1 of 2

| **Section/topic** | **#** | **Checklist item** | **Reported paragraph # (within section)** |
| --- | --- | --- | --- |
| Risk of bias across studies | 15 | Specify any assessment of risk of bias that may affect the cumulative evidence (e.g., publication bias, selective reporting within studies). | **4** |
| Additional analyses | 16 | Describe methods of additional analyses (e.g., sensitivity or subgroup analyses, meta-regression), if done, indicating which were pre-specified. | **4** |
| **RESULTS** | | |  |
| Study selection | 17 | Give numbers of studies screened, assessed for eligibility, and included in the review, with reasons for exclusions at each stage, ideally with a flow diagram. | **1, Appendix Figure 1** |
| Study characteristics | 18 | For each study, present characteristics for which data were extracted (e.g., study size, PICOS, follow-up period) and provide the citations. | **2,** **Appendix Table 1,2,3** |
| Risk of bias within studies | 19 | Present data on risk of bias of each study and, if available, any outcome level assessment (see item 12). | **N/A** |
| Results of individual studies | 20 | For all outcomes considered (benefits or harms), present, for each study: (a) simple summary data for each intervention group (b) effect estimates and confidence intervals, ideally with a forest plot. | **Figure 1,2** |
| Synthesis of results | 21 | Present results of each meta-analysis done, including confidence intervals and measures of consistency. | **3,8,9, Figure 1-2** |
| Risk of bias across studies | 22 | Present results of any assessment of risk of bias across studies (see Item 15). | **5, Appendix Figure 2** |
| Additional analysis | 23 | Give results of additional analyses, if done (e.g., sensitivity or subgroup analyses, meta-regression [see Item 16]). | **4, 6,7,Table 1-2** |
| **DISCUSSION** | | |  |
| Summary of evidence | 24 | Summarize the main findings including the strength of evidence for each main outcome; consider their relevance to key groups (e.g., healthcare providers, users, and policy makers). | **1-3,5** |
| Limitations | 25 | Discuss limitations at study and outcome level (e.g., risk of bias), and at review-level (e.g., incomplete retrieval of identified research, reporting bias). | **4** |
| Conclusions | 26 | Provide a general interpretation of the results in the context of other evidence, and implications for future research. | **6** |
| **FUNDING** | | |  |
| Funding | 27 | Describe sources of funding for the systematic review and other support (e.g., supply of data); role of funders for the systematic review. | **See submission form** |

*From:*  Moher D, Liberati A, Tetzlaff J, Altman DG, The PRISMA Group (2009). Preferred Reporting Items for Systematic Reviews and Meta-Analyses: The PRISMA Statement. PLoS Med 6(6): e1000097. doi:10.1371/journal.pmed1000097

For more information, visit: **www.prisma-statement.org**.

Page 2 of 2

**Appendix Figure 1.** Flowchart of the selection of studies included in meta-analysis.


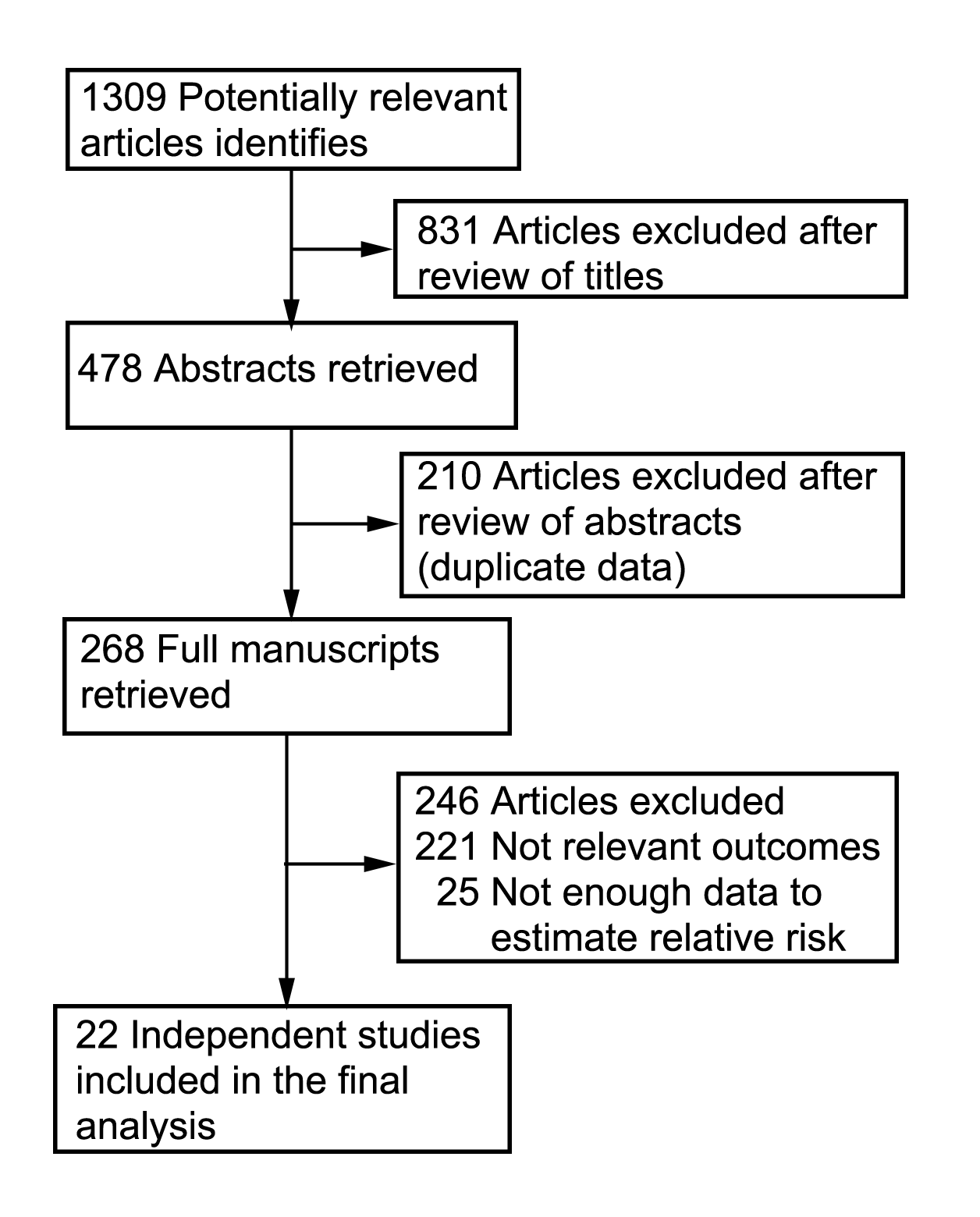


**Appendix Figure 2.** Funnel plots showing association of death from any cause with digoxin in patients with atrial fibrillation.


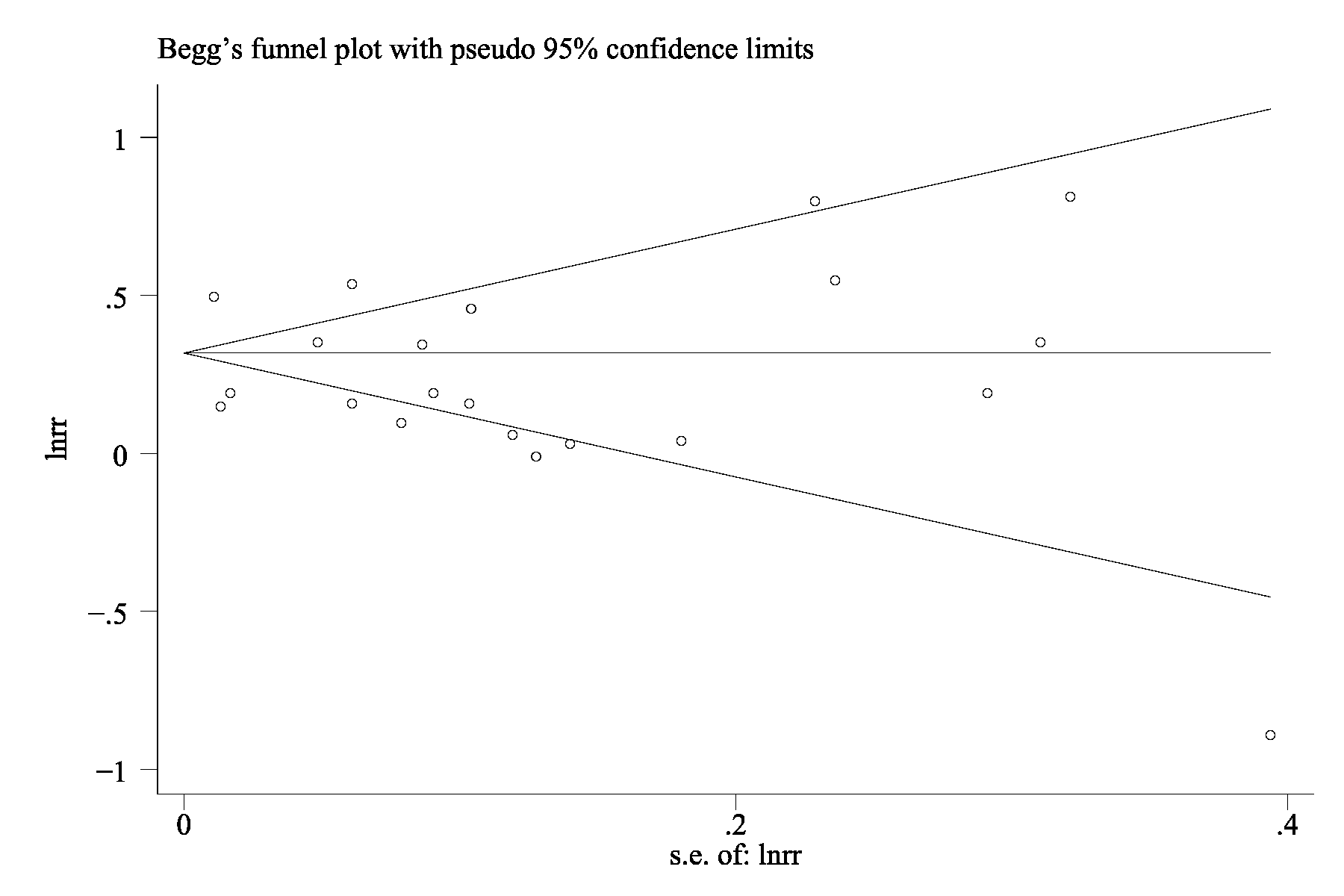


**Appendix Figure 3.** Forest plot showing relative risks of heart failure hospitalization, acute myocardial infarction and all cause hospitalization associated with digoxin therapy in patients with atrial fibrillation.

**Appendix Table 1. Summary of available studies included in the present meta-analysis.**

| Study | Study type | Country | Average Follow-up (months) | Mean age | Digoxin use defined as | Outcomes | Adjustment |
| --- | --- | --- | --- | --- | --- | --- | --- |
| Wang J, 2013 ^1^ | Prospective cohort | China | 12 | 68.5 | Baseline use | Death from any cause | Age, gender, smoking, race, alcohol, body mass index, hypertension, diabetes, heart failure, coronary heart disease, propensity scores |
| Fauchier L, 2009 ^2^ | Prospective cohort | France | 29.4 | 74 | Baseline use | Death from any cause | Age, gender, hypertension, diabetes, heart failure, coronary heart disease, stroke |
| SPORTIF III and V studies, 2007 ^3^ | Post-hoc analysis of RCT | Multi-national | 17.4 | 71 | Baseline use | Death from any cause, stroke | Age, gender, smoking, race, alcohol, body mass index, hypertension, diabetes, heart failure, coronary heart disease |
| Dhaliwal A, 2008 ^4^ | Retrospective cohort | USA | 10.1 | 68 | Baseline use | Death from any cause, heart failure hospitalization | Age, heart failure |
| RISK-HIA, 2007 ^5^ | Prospective cohort | Sweden | 12 | 75 | Baseline use | Death from any cause, heart failure hospitalization | Age, gender, smoking, hypertension, diabetes, heart failure, propensity scores |
| ATRIA-CVRN study, 2015 ^6^ | Retrospective cohort | USA | 14 | 70.7 | Incident use | Death from any cause, all cause hospitalization | Age, gender, smoking, race, alcohol, body mass index, hypertension, diabetes, heart failure, coronary heart disease, propensity scores |
| SCAF study, 2009 ^7^ | Retrospective cohort | Sweden | 55.2 | 75 | Baseline use | Death from any cause, stroke, heart failure hospitalization, myocardial infarction | Age, gender, smoking, race, alcohol, body mass index, hypertension, diabetes, heart failure, coronary heart disease, propensity scores |
| RACE II study, 2014 ^8^ | Post-hoc analysis of RCT | Netherlands | 34.8 | 68 | Baseline use | Death from any cause, cardiovascular mortality, all cause hospitalization, stroke, heart failure hospitalization, myocardial infarction | Age, gender, hypertension, heart failure |
| Georgiopoulou V, 2009 ^9^ | Retrospective cohort | USA | 27 | 52 | Baseline use | Death from any cause, heart failure hospitalization | Age, gender, race, hypertension, diabetes, heart failure, coronary heart disease, propensity scores |
| Pastori D, 2015 ^10^ | Prospective cohort | Italy | 33.2 | 73 | Baseline use | Death from any cause, cardiovascular death | Age, gender, body mass index, hypertension, diabetes, heart failure, coronary heart disease, propensity scores |
| LIFE study, 2015 ^11^ | Retrospective cohort | Multi-national | 56.4 | 70 | Incident use | Death from any cause, cardiovascular death, sudden cardiac death | Age, diabetes, cholesterol, heart failure, coronary heart disease, stroke, propensity scores |
| ORBIT-AF, 2014 ^12^ | Prospective cohort | USA | 24 | 76 | Baseline use; incident use | Death from any cause, all cause hospitalization | Age, gender, smoking, cholesterol, race, alcohol, body mass index, hypertension, diabetes, heart failure, coronary heart disease, stroke, propensity scores |
| ROCKRT-AF study, 2015 ^13^ | Post-hoc analysis of RCT | Multi-national | 23.6 | 73 | Baseline use; incident use | Death from any cause, cardiovascular death, all cause hospitalization, stroke, heart failure hospitalization, myocardial infarction, sudden cardiac death | Age, gender, smoking, cholesterol, race, alcohol, body mass index, hypertension, diabetes, heart failure, coronary heart disease, stroke |
| Chang S, 2013 ^14^ | Retrospective cohort | China | 43.6 | 74.3 | Baseline use | Stroke | Age, gender, cholesterol, hypertension, diabetes, coronary heart disease |
| Chao T, 2014 ^15^ | Retrospective cohort | China | 51.8 | 67.8 | Baseline use | Death from any cause, stroke | Age, gender, smoking, hypertension, diabetes, heart failure, coronary heart disease |
| Frasure-Smith N, 2009 ^16^ | Post-hoc analysis of RCT | Multi-national | 39 | 66 | Baseline use | Death from any cause, cardiovascular death, arrhythmic death | Age, gender, race, cholesterol, alcohol, body mass index, hypertension, diabetes, heart failure, coronary heart disease, stroke |
| TREAT-AF study, 2014 ^17^ | Retrospective cohort | USA | 34.6 | 70 | Incident use | Death from any cause | Age, gender, cholesterol, body mass index, hypertension, diabetes, heart failure, coronary heart disease, stroke, propensity scores |
| PALLAS trial, 2014 ^18^ | Post-hoc analysis of RCT | Multi-national | 6 | 75 | Baseline use | Death from any cause, cardiovascular mortality, arrhythmic death | Age, gender, hypertension, heart failure, coronary heart disease |
| AFFRIM trial, 2013 ^19^ | Post-hoc analysis of RCT | USA | 42 | 70 | Incident use | Death from any cause, cardiovascular death, all cause hospitalization, arrhythmic death | Age, gender, race, cholesterol, alcohol, body mass index, hypertension, diabetes, heart failure, coronary heart disease, stroke, propensity scores |
| AFBAR study, 2014 ^20^ | Prospective cohort | Spain | 35.6 | 74.9 | Baseline use | Death from any cause, all cause hospitalization | Age, gender, race, cholesterol, alcohol, body mass index, hypertension, diabetes, heart failure, coronary heart disease, stroke |
| Shah M, 2014 ^21^ | Retrospective cohort | Canada | 48 | 79 | Baseline use | Death from any cause | Age, gender, body mass index, hypertension, diabetes, heart failure, coronary heart disease, stroke, propensity scores |
| Hvidtfelet M, 2013 ^22^ | Retrospective cohort | Denmark | 56.4 | 74 | Baseline use | Death from any cause, cardiovascular death, stroke | Age, gender, race, cholesterol, alcohol, body mass index, hypertension, diabetes, heart failure, coronary heart disease, stroke |

**References:**

**1.** Wang J, Yang YM, Zhu J, et al. [Analysis of risk factors for all cause-mortality in Chinese emergency atrial fibrillation patients]. *Zhonghua Yi Xue Za Zhi.* 2013;93(36):2871-2875.

**2.** Fauchier L, Grimard C, Pierre B, et al. Comparison of beta blocker and digoxin alone and in combination for management of patients with atrial fibrillation and heart failure. *Am J Cardiol.* 2009;103(2):248-254.

**3.** Gjesdal K, Feyzi J, Olsson SB. Digitalis: a dangerous drug in atrial fibrillation? An analysis of the SPORTIF III and V data. *Heart.* 2008;94(2):191-196.

**4.** Dhaliwal AS, Bredikis A, Habib G, Carabello BA, Ramasubbu K, Bozkurt B. Digoxin and clinical outcomes in systolic heart failure patients on contemporary background heart failure therapy. *Am J Cardiol.* 2008;102(10):1356-1360.

**5.** Hallberg P, Lindback J, Lindahl B, Stenestrand U, Melhus H. Digoxin and mortality in atrial fibrillation: a prospective cohort study. *Eur J Clin Pharmacol.* 2007;63(10):959-971.

**6.** Freeman JV, Reynolds K, Fang M, et al. Digoxin and risk of death in adults with atrial fibrillation: the ATRIA-CVRN study. *Circ Arrhythm Electrophysiol.* 2015;8(1):49-58.

**7.** Friberg L, Hammar N, Rosenqvist M. Digoxin in atrial fibrillation: report from the Stockholm Cohort study of Atrial Fibrillation (SCAF). *Heart.* 2010;96(4):275-280.

**8.** Mulder BA, Van Veldhuisen DJ, Crijns HJ, et al. Digoxin in patients with permanent atrial fibrillation: data from the RACE II study. *Heart Rhythm.* 2014;11(9):1543-1550.

**9.** Georgiopoulou VV, Kalogeropoulos AP, Giamouzis G, et al. Digoxin therapy does not improve outcomes in patients with advanced heart failure on contemporary medical therapy. *Circ Heart Fail.* 2009;2(2):90-97.

**10.** Pastori D, Farcomeni A, Bucci T, et al. Digoxin treatment is associated with increased total and cardiovascular mortality in anticoagulated patients with atrial fibrillation. *Int J Cardiol.* 2015;180:1-5.

**11.** Okin PM, Hille DA, Wachtell K, et al. Digoxin use and risk of mortality in hypertensive patients with atrial fibrillation. *J Hypertens.* 2015;33(7):1480-1486.

**12.** Allen LA, Fonarow GC, Simon DN, et al. Digoxin Use and Subsequent Outcomes Among Patients in a Contemporary Atrial Fibrillation Cohort. *J Am Coll Cardiol.* 2015;65(25):2691-2698.

**13.** Washam JB, Stevens SR, Lokhnygina Y, et al. Digoxin use in patients with atrial fibrillation and adverse cardiovascular outcomes: a retrospective analysis of the Rivaroxaban Once Daily Oral Direct Factor Xa Inhibition Compared with Vitamin K Antagonism for Prevention of Stroke and Embolism Trial in Atrial Fibrillation (ROCKET AF). *Lancet.* 2015;385(9985):2363-2370.

**14.** Chang SS, Chang KC, Wang YC, et al. Digoxin use is associated with increased risk of stroke in patients with non-valvular atrial fibrillation--a nationwide population-based cohort study. *Int J Cardiol.* 2013;169(2):e26-e27.

**15.** Chao TF, Liu CJ, Chen SJ, et al. Does digoxin increase the risk of ischemic stroke and mortality in atrial fibrillation? A nationwide population-based cohort study. *Can J Cardiol.* 2014;30(10):1190-1195.

**16.** Frasure-Smith N, Lesperance F, Habra M, et al. Elevated depression symptoms predict long-term cardiovascular mortality in patients with atrial fibrillation and heart failure. *Circulation.* 2009;120(2):134-140, 3p-140p.

**17.** Turakhia MP, Santangeli P, Winkelmayer WC, et al. Increased mortality associated with digoxin in contemporary patients with atrial fibrillation: findings from the TREAT-AF study. *J Am Coll Cardiol.* 2014;64(7):660-668.

**18.** Hohnloser SH, Halperin JL, Camm AJ, Gao P, Radzik D, Connolly SJ. Interaction between digoxin and dronedarone in the PALLAS trial. *Circ Arrhythm Electrophysiol.* 2014;7(6):1019-1025.

**19.** Whitbeck MG, Charnigo RJ, Khairy P, et al. Increased mortality among patients taking digoxin--analysis from the AFFIRM study. *Eur Heart J.* 2013;34(20):1481-1488.

**20.** Rodriguez-Manero M, Otero-Ravina F, Garcia-Seara J, et al. Outcomes of a contemporary sample of patients with atrial fibrillation taking digoxin: results from the AFBAR study. *Rev Esp Cardiol (Engl Ed).* 2014;67(11):890-897.

**21.** Shah M, Avgil TM, Jackevicius CA, Essebag V, Behlouli H, Pilote L. Relation of digoxin use in atrial fibrillation and the risk of all-cause mortality in patients >/=65 years of age with versus without heart failure. *Am J Cardiol.* 2014;114(3):401-406.

**22.** Hvidtfeldt MW, Andersson C, Mikkelsen A, Gislason G, Torp-Pedersen C, Hansen ML. Safety in rate control medication in patients with atrial fibrillation: a nationwide study. *Eur Heart J.* 2013;341:750.

**Appendix Table 2. Publications reporting data on digoxin dosing and/or plasma levels.**

| Study, | Patient number | Mean digoxin dose (mg) | Mean serum digoxin concentration  (ng/mL) |
| --- | --- | --- | --- |
| ATRIA-CVRN study, 2014 ^1^ | 14,787 | 0.164 | 0.964 |
| RACE II study, 2014 ^2^ | 614 | 0.25 | No data |
| Pastori, 2015 ^3^ | 815 | 0.126 | No data |
| Georgiopoulou V, 2009 ^4^ | 455 | 0.13 | 0.75 |
| PALLAS trial, 2014 ^5^ | 3,236 | No data | 0.9 |

**References:**

**1.** Freeman JV, Reynolds K, Fang M, et al. Digoxin and risk of death in adults with atrial fibrillation: the ATRIA-CVRN study. *Circ Arrhythm Electrophysiol.* 2015;8(1):49-58.

**2.** Mulder BA, Van Veldhuisen DJ, Crijns HJ, et al. Digoxin in patients with permanent atrial fibrillation: data from the RACE II study. *Heart Rhythm.* 2014;11(9):1543-1550.

**3.** Pastori D, Farcomeni A, Bucci T, et al. Digoxin treatment is associated with increased total and cardiovascular mortality in anticoagulated patients with atrial fibrillation. *Int J Cardiol.* 2015;180:1-5.

**4.** Georgiopoulou VV, Kalogeropoulos AP, Giamouzis G, et al. Digoxin therapy does not improve outcomes in patients with advanced heart failure on contemporary medical therapy. *Circ Heart Fail.* 2009;2(2):90-97.

**5.** Hohnloser SH, Halperin JL, Camm AJ, Gao P, Radzik D, Connolly SJ. Interaction between digoxin and dronedarone in the PALLAS trial. *Circ Arrhythm Electrophysiol.* 2014;7(6):1019-1025.

**Appendix Table 3. Quality of included studies.**

| Study | Aim^*^ | Inclusion^†^ | Data^‡^ | Measurement§ | Bias^‖^ | Time^¶^ | Loss^#^ | Size^**^ | Control^††^ | Contemporary^‡‡^ | Factor^§§^ | Analysi^‖‖^ | Total |
| --- | --- | --- | --- | --- | --- | --- | --- | --- | --- | --- | --- | --- | --- |
| Wang J, 2013 ^1^ | 2 | 2 | 2 | 2 | 0 | 2 | 2 | 1 | 2 | 2 | 1 | 2 | 20 |
| Fauchier L, 2009 ^2^ | 2 | 2 | 2 | 2 | 0 | 2 | 0 | 1 | 2 | 2 | 1 | 2 | 19 |
| SPORTIF III and V studies, 2007 ^3^ | 2 | 2 | 2 | 2 | 2 | 2 | 0 | 0 | 2 | 2 | 1 | 2 | 19 |
| Dhaliwal A, 2008 ^4^ | 2 | 2 | 2 | 2 | 0 | 2 | 0 | 1 | 2 | 2 | 1 | 2 | 18 |
| RISK-HIA, 2007 ^5^ | 2 | 1 | 2 | 2 | 0 | 2 | 0 | 1 | 2 | 2 | 2 | 2 | 18 |
| ATRIA-CVRN study, 2015 ^6^ | 2 | 2 | 2 | 2 | 0 | 2 | 0 | 1 | 2 | 2 | 2 | 2 | 19 |
| SCAF study, 2009 ^7^ | 2 | 1 | 2 | 2 | 0 | 2 | 0 | 1 | 2 | 2 | 2 | 2 | 18 |
| RACE II study, 2014 ^8^ | 2 | 2 | 2 | 2 | 1 | 2 | 2 | 1 | 2 | 2 | 1 | 1 | 20 |
| Georgiopoulou V, 2009 ^9^ | 2 | 2 | 2 | 2 | 0 | 2 | 0 | 1 | 2 | 2 | 2 | 2 | 19 |
| Pastori D, 2015 ^10^ | 2 | 2 | 2 | 2 | 2 | 2 | 0 | 2 | 2 | 2 | 2 | 2 | 22 |
| LIFE study, 2015 ^11^ | 2 | 1 | 2 | 2 | 2 | 2 | 0 | 1 | 2 | 2 | 2 | 2 | 20 |
| ORBIT-AF, 2014 ^12^ | 2 | 2 | 2 | 2 | 0 | 2 | 2 | 2 | 2 | 2 | 2 | 2 | 22 |
| ROCKRT-AF study, 2015 ^13^ | 2 | 2 | 2 | 2 | 2 | 2 | 0 | 1 | 2 | 2 | 2 | 2 | 21 |
| Chang S, 2013 ^14^ | 2 | 2 | 2 | 2 | 0 | 2 | 0 | 1 | 2 | 2 | 0 | 2 | 17 |
| Chao T, 2014 ^15^ | 2 | 2 | 2 | 2 | 0 | 2 | 0 | 1 | 2 | 2 | 1 | 2 | 18 |
| Frasure-Smith N, 2009 ^16^ | 2 | 2 | 2 | 2 | 2 | 2 | 2 | 1 | 2 | 2 | 1 | 2 | 22 |
| TREAT-AF study, 2014 ^17^ | 2 | 2 | 2 | 2 | 0 | 2 | 0 | 1 | 2 | 2 | 2 | 2 | 19 |
| PALLAS trial, 2014 ^18^ | 2 | 2 | 2 | 2 | 2 | 2 | 2 | 1 | 2 | 2 | 2 | 2 | 23 |
| AFFRIM trial, 2013 ^19^ | 2 | 2 | 2 | 2 | 2 | 2 | 2 | 1 | 2 | 2 | 2 | 2 | 23 |
| AFBAR study, 2014 ^20^ | 2 | 1 | 2 | 2 | 0 | 2 | 0 | 1 | 2 | 2 | 1 | 2 | 17 |
| Shah M, 2014 ^21^ | 2 | 2 | 2 | 2 | 0 | 2 | 0 | 1 | 2 | 2 | 2 | 2 | 19 |
| Hvidtfelet M, 2013 ^22^ | 2 | 1 | 2 | 2 | 0 | 2 | 0 | 1 | 2 | 2 | 0 | 2 | 17 |

Methodologic quality was assessed using the Methodological Index for Non-Randomized Studies.

0 =not reported; 1= inadequately reported; 2=adequately reported.

^*^ Aim of study.

^†^ Inclusion of consecutive patients and participation rate.

^‡^ Prospective data collection.

^§^ End points appropriate to aim of study.

^‖^Unbiased assessment of study end points.

^¶^ Appropriateness of follow-up time after diagnosis.

^# I^nclusion of loss to follow-up.

^**^ Prospective calculation of study size.

^††^ Comparable control group.

^‡‡^ Contemporary control groups.

^§§^ Baseline equivalence of groups on several factors.

^‖‖^Adequate statistical analysis.

**References:**

**1.** Wang J, Yang YM, Zhu J, et al. [Analysis of risk factors for all cause-mortality in Chinese emergency atrial fibrillation patients]. *Zhonghua Yi Xue Za Zhi.* 2013;93(36):2871-2875.

**2.** Fauchier L, Grimard C, Pierre B, et al. Comparison of beta blocker and digoxin alone and in combination for management of patients with atrial fibrillation and heart failure. *Am J Cardiol.* 2009;103(2):248-254.

**3.** Gjesdal K, Feyzi J, Olsson SB. Digitalis: a dangerous drug in atrial fibrillation? An analysis of the SPORTIF III and V data. *Heart.* 2008;94(2):191-196.

**4.** Dhaliwal AS, Bredikis A, Habib G, Carabello BA, Ramasubbu K, Bozkurt B. Digoxin and clinical outcomes in systolic heart failure patients on contemporary background heart failure therapy. *Am J Cardiol.* 2008;102(10):1356-1360.

**5.** Hallberg P, Lindback J, Lindahl B, Stenestrand U, Melhus H. Digoxin and mortality in atrial fibrillation: a prospective cohort study. *Eur J Clin Pharmacol.* 2007;63(10):959-971.

**6.** Freeman JV, Reynolds K, Fang M, et al. Digoxin and risk of death in adults with atrial fibrillation: the ATRIA-CVRN study. *Circ Arrhythm Electrophysiol.* 2015;8(1):49-58.

**7.** Friberg L, Hammar N, Rosenqvist M. Digoxin in atrial fibrillation: report from the Stockholm Cohort study of Atrial Fibrillation (SCAF). *Heart.* 2010;96(4):275-280.

**8.** Mulder BA, Van Veldhuisen DJ, Crijns HJ, et al. Digoxin in patients with permanent atrial fibrillation: data from the RACE II study. *Heart Rhythm.* 2014;11(9):1543-1550.

**9.** Georgiopoulou VV, Kalogeropoulos AP, Giamouzis G, et al. Digoxin therapy does not improve outcomes in patients with advanced heart failure on contemporary medical therapy. *Circ Heart Fail.* 2009;2(2):90-97.

**10.** Pastori D, Farcomeni A, Bucci T, et al. Digoxin treatment is associated with increased total and cardiovascular mortality in anticoagulated patients with atrial fibrillation. *Int J Cardiol.* 2015;180:1-5.

**11.** Okin PM, Hille DA, Wachtell K, et al. Digoxin use and risk of mortality in hypertensive patients with atrial fibrillation. *J Hypertens.* 2015;33(7):1480-1486.

**12.** Allen LA, Fonarow GC, Simon DN, et al. Digoxin Use and Subsequent Outcomes Among Patients in a Contemporary Atrial Fibrillation Cohort. *J Am Coll Cardiol.* 2015;65(25):2691-2698.

**13.** Washam JB, Stevens SR, Lokhnygina Y, et al. Digoxin use in patients with atrial fibrillation and adverse cardiovascular outcomes: a retrospective analysis of the Rivaroxaban Once Daily Oral Direct Factor Xa Inhibition Compared with Vitamin K Antagonism for Prevention of Stroke and Embolism Trial in Atrial Fibrillation (ROCKET AF). *Lancet.* 2015;385(9985):2363-2370.

**14.** Chang SS, Chang KC, Wang YC, et al. Digoxin use is associated with increased risk of stroke in patients with non-valvular atrial fibrillation--a nationwide population-based cohort study. *Int J Cardiol.* 2013;169(2):e26-e27.

**15.** Chao TF, Liu CJ, Chen SJ, et al. Does digoxin increase the risk of ischemic stroke and mortality in atrial fibrillation? A nationwide population-based cohort study. *Can J Cardiol.* 2014;30(10):1190-1195.

**16.** Frasure-Smith N, Lesperance F, Habra M, et al. Elevated depression symptoms predict long-term cardiovascular mortality in patients with atrial fibrillation and heart failure. *Circulation.* 2009;120(2):134-140, 3p-140p.

**17.** Turakhia MP, Santangeli P, Winkelmayer WC, et al. Increased mortality associated with digoxin in contemporary patients with atrial fibrillation: findings from the TREAT-AF study. *J Am Coll Cardiol.* 2014;64(7):660-668.

**18.** Hohnloser SH, Halperin JL, Camm AJ, Gao P, Radzik D, Connolly SJ. Interaction between digoxin and dronedarone in the PALLAS trial. *Circ Arrhythm Electrophysiol.* 2014;7(6):1019-1025.

**19.** Whitbeck MG, Charnigo RJ, Khairy P, et al. Increased mortality among patients taking digoxin--analysis from the AFFIRM study. *Eur Heart J.* 2013;34(20):1481-1488.

**20.** Rodriguez-Manero M, Otero-Ravina F, Garcia-Seara J, et al. Outcomes of a contemporary sample of patients with atrial fibrillation taking digoxin: results from the AFBAR study. *Rev Esp Cardiol (Engl Ed).* 2014;67(11):890-897.

**21.** Shah M, Avgil TM, Jackevicius CA, Essebag V, Behlouli H, Pilote L. Relation of digoxin use in atrial fibrillation and the risk of all-cause mortality in patients >/=65 years of age with versus without heart failure. *Am J Cardiol.* 2014;114(3):401-406.

**22.** Hvidtfeldt MW, Andersson C, Mikkelsen A, Gislason G, Torp-Pedersen C, Hansen ML. Safety in rate control medication in patients with atrial fibrillation: a nationwide study. *Eur Heart J.* 2013;341:750.
